# Rehabilitation Outcomes Following Surgical Management of Lower-Limb Soft Tissue Sarcomas: Insights from Gait Analysis

**DOI:** 10.3390/jcm14176061

**Published:** 2025-08-27

**Authors:** Marco Germanotta, Francesca Falchini, Arianna Pavan, Stefania Lattanzi, Laura Cortellini, Beniamino Brunetti, Stefania Tenna, Alice Valeri, Chiara Pagnoni, Roberto Passa, Michela Angelucci, Bruno Vincenzi, Rossana Alloni, Irene Giovanna Aprile, Sergio Valeri

**Affiliations:** 1 IRCCS Fondazione Don Carlo Gnocchi, 50143 Florence, Italy; mgermanotta@dongnocchi.it (M.G.); apavan@dongnocchi.it (A.P.); slattanzi@dongnocchi.it (S.L.); lcortellini@dongnocchi.it (L.C.); iaprile@dongnocchi.it (I.G.A.); 2 Operative Research Unit of Plastic-Reconstructive and Aesthetic Surgery, Fondazione Policlinico Universitario Campus Bio-Medico, 00128 Rome, Italy; b.brunetti@policlinicocampus.it (B.B.); s.tenna@policlinicocampus.it (S.T.); 3 Operative Research Unit of Plastic-Reconstructive and Aesthetic Surgery, Università Campus Bio-Medico, 00128 Rome, Italy; alice.valeri@alcampus.it; 4 Operative Research Unit, Soft-Tissue Sarcomas Surgery Department, Fondazione Policlinico Universitario Campus Bio-Medico, 00128 Rome, Italy; c.pagnoni@policlinicocampus.it (C.P.); r.passa@policlinicocampus.it (R.P.); michela.angelucci@unicampus.it (M.A.); r.alloni@policlinicocampus.it (R.A.); s.valeri@policlinicocampus.it (S.V.); 5 Operative Research Unit of Medical Oncology, Fondazione Policlinico Universitario Campus Bio-Medico, 00128 Rome, Italy; b.vincenzi@policlinicocampus.it

**Keywords:** soft tissue sarcoma, rehabilitation, surgery, gait analysis

## Abstract

**Background:** Soft tissue sarcomas (STSs) are rare and heterogeneous malignancies requiring a multidisciplinary approach to diagnosis and treatment. Advances in surgical techniques, chemotherapy, and radiotherapy have improved survival rates but often result in significant functional impairments, particularly in patients undergoing limb-sparing procedures. Rehabilitation is crucial for restoring mobility and independence, with recent studies emphasizing the importance of personalized rehabilitation protocols tailored to specific surgical interventions. Quantitative assessments, such as 3D motion capture and surface electromyography, provide objective insights into gait performance and motor function, enabling more precise rehabilitation strategies to optimize recovery. **Methods:** This study evaluated gait performance in 21 patients with lower-limb impairment following limb-sparing surgery for STS. Patients underwent two instrumented gait assessments using marker-based 3D motion capture and surface electromyography to measure spatiotemporal gait parameters, joint kinematics, and muscle activity. Independence in the activity of daily living was assessed with the modified Barthel Index in both timepoints. **Results:** Following rehabilitation, patients demonstrated significant improvements in functional independence, as reflected by an increase in the modified Barthel Index (*p* < 0.001). Gait analysis revealed increased walking speed, stride length, cadence, and improved joint range of motion at the hip, knee, and ankle, though electromyographic analysis showed no statistically significant differences in muscle activation patterns or co-contraction indices. **Conclusions:** These findings underscore the importance of a rehabilitation programs personalized on gait strategies. A deeper understanding of motor adaptations based on sarcoma location and surgical approach could further refine rehabilitation protocols, ultimately enhancing patient outcomes and quality of life.

## 1. Introduction

Soft tissue sarcomas (STSs) are a rare and diverse group of malignancies, accounting for about 1% of adult cancers [1]. These tumors can arise in various anatomical regions, including the trunk, the extremities, and the abdomen. The limbs are the most common site, representing approximately 50–60% of cases [2], with the lower limbs being involved at a rate nearly three times that of the upper limbs [3]. The World Health Organization (WHO) recognizes over 100 distinct histological and molecular subtypes of soft tissue sarcomas, each demonstrating diverse clinical behaviors [4]. This complexity necessitates a thorough diagnostic process, including biopsies and consultations among specialists such as surgeons, pathologists, oncologists, and radiologists, to devise individualized treatment plans [5]. Given the rarity of these tumors, the expertise required for their management is often concentrated in specialized centers that facilitate collaborative, multidisciplinary care [1].

Treatment approaches for STSs are tailored based on factors such as histology of the tumor, extent, aggressiveness, and age of the patient. Surgical intervention, typically involving wide resection, is the primary treatment for localized disease and aims to achieve negative tumor excision margins [6]. It is often supplemented by radiotherapy [7] or chemotherapy, either before or after the resection [8], depending on the tumor’s characteristics. Limb preservation is the primary objective, though amputation may be necessary in a minority of cases where the disease is extensive [9]. In Europe, patients with STS have an estimated 5-year relative survival of 58% and an overall survival of about 50% [10]. Consequently, current guidelines advocate for excision with clear oncologic margins based on the tumor’s histological subtype [11].

Advances in the management of STSs have led to significant improvements in patient survival rates, but these successes have been accompanied by an increase in mid- to long-term disabilities. The aggressive nature of surgical interventions, which may require extensive tissue resection or reconstruction, commonly leads to deficits in motor function and mobility [12]. These impairments can significantly affect the patient’s quality of life [13] and reduce the likelihood of returning to work [14]. In addition, chronic pain is a prevalent consequence following treatment, further diminishing the ability to engage in routine activities and contributing to a negative impact on psychological health. In addition to neuropathic pain, sensory loss, impaired proprioception, and distal muscle weakness may compromise balance control, increasing the risk of falls and reducing functional mobility [14].

This complex interplay emphasizes the importance of multidisciplinary rehabilitation after surgery, addressing the long-term physical, emotional, and social challenges faced by STS patients [15]. In the review of Andrews et al. [14], the importance of a multidisciplinary rehabilitation plan, made by a rehabilitation specialist to determine appropriate interventions, is emphasized. In a very recent study [16], our research group presented a customized rehabilitation protocol specifically designed to accommodate the unique surgical approaches of individual patients. The importance of rehabilitation across the entire continuum of care was also recently emphasized by the British Sarcoma Group through the publication of a consensus-based guideline [17].

This consensus-based guideline [17] also emphasizes the urgent need to develop innovative objective measures of physical function to advance evidence-based clinical practices. From this perspective, laboratory-based and portable systems that evaluate gait kinematics, postural control, and deviations offer valuable insights into balance and gait, providing key outcomes essential for identifying optimal approaches for individuals and delivering evidence-based rehabilitation treatments. By providing measurable outcomes, these instruments play a pivotal role in guiding clinical decisions, monitoring progress, and optimizing treatment strategies to achieve the best possible functional recovery for patients.

Clinical use of qualitative gait analyses is common in cases of lower-limb impairment sarcoma, since gait pattern alterations often result from significant muscle or nerve resections [18]. For instance, complete quadriceps group resections can lead to compensatory strategies such as back-knee gait [12,19]. However, few studies quantify balance and gait in patients with lower extremity impairment after sarcoma surgery, using instrumented assessment [20], to identify subtle, progressive changes in gait in patients undergoing rehabilitation for limb salvage [19]. Two case studies, reported by Tanaka et al. [21] and Kubota et al. [22], based on instrumented gait analysis, showed compensatory adaptations—mainly increased hamstring and gastrocnemius activity, as well as altered hip and ankle kinematics—highlighting the need for targeted muscle strengthening in rehabilitation. Recently, the feasibility of utilizing accelerometer-based body-worn monitor assessments [23] or markerless motion capture [24] to assess balance and gait was explored in a heterogeneous cohort group of patients with bone tumors and soft tissue sarcomas. However, to the best of our knowledge, no studies have been published that specifically analyze the effects of rehabilitation interventions in a sample of patients with lower-limb impairment due to soft tissue sarcoma using marker-based 3D motion capture systems, which are widely regarded as the gold standard for motion analysis. This gap in research highlights the potential value of incorporating advanced motion capture technology to enhance our understanding of rehabilitation outcomes and to better tailor interventions for improved patient outcomes.

Building upon our results described above [16], the current study aims to further investigate the effects of previously developed tailored rehabilitation intervention, including a comprehensive quantitative analysis of gait performance, utilizing a marker-based 3D motion capture system alongside surface electromyography.

## 2. Materials and Methods

### 2.1. Study Design

This study analyzes data collected from patients with lower-limb impairment due to limb-sparing surgery for STS performed at Fondazione Policlinico Universitario Campus Bio-Medico and consequent rehabilitation treatment at the Santa Maria della Provvidenza Center of the Fondazione Don Carlo Gnocchi Onlus in Rome.

Informed consent was obtained from all participants. The study was approved by the Ethics Committee of the Fondazione Policlinico Universitario Campus Bio-Medico (Protocol number PAR 77.22 OSS) and by the Ethics Committee Lazio 1 (Protocol number 420/CE Lazio 1). It was registered on ClinicalTrials.gov (ID NCT06282237). This study presents data from a subgroup of patients enrolled in a previous study, the clinical results of which have been reported elsewhere [16].

### 2.2. Subjects

In the study, we included adult patients with soft tissue sarcoma of the lower extremities or retroperitoneum with femoral nerve injury who underwent demolitive surgery with curative intent. We excluded patients with recurrent tumors, metastatic disease, palliative surgery, or amputations. Based on clinical requirements, various surgical approaches were employed. Specifically, some patients underwent reconstructive procedures utilizing techniques such as demolition surgery, free functional muscle transfer, free flap, and local perforator flap. At admission and discharge from the rehabilitation ward, patients’ abilities in activities of daily living were assessed using the modified Barthel Index [25].

### 2.3. Rehabilitation Protocol

All patients participated in two 50-min rehabilitation sessions per day, conducted 6 days per week. The rehabilitation program included both conventional and robotic approaches, designed to enhance strength, balance, proprioception, and range of motion while facilitating the restoration of ambulation. The specific rehabilitation protocols were customized based on the surgical procedures performed. The key rehabilitation steps include postural steps and bed–wheelchair transfers, verticalization, ambulation without weight bearing on the operated limb or with partial bearing, and ambulation with gradual weight bearing with or without a brace, with a timeline from the first to the latter selected according to the surgical approaches (demolition surgery, local perforator flaps, free flap, or free functional muscle transfer). More information on the rehabilitation protocol was previously reported [16].

### 2.4. Instrumented Assessment

Among patients recruited for the study, 36 patients underwent an instrumented evaluation of gait once able to walk (T0), with or without an aid. Of those, 21 patients were also re-evaluated using gait analysis at discharge (T1) and included in the present study. The examination was conducted at the Movement Analysis Laboratory of the Fondazione Don Carlo Gnocchi in Rome. Patients were instructed to perform barefoot overground walking trails along a 10 m walkway at a self-selected speed. The use of a single forearm crutch during the assessment was allowed, if required, according to each patient’s gait capabilities. The same aid was then consistently utilized during the T1 evaluation to ensure consistency in experimental conditions.

Data acquisition was performed using an 8-camera stereophotogrammetric system (Smart D500, BTS Bioengineering, Milan, Italy). Before each acquisition session, the cameras were calibrated following the standard procedures described by the manufacturer. Twenty-two retro-reflective markers were placed on anatomical landmarks following the standardized protocol described by Davis et al. [26]. Kinematic data were collected at 100 Hz, and kinetic data were collected using two ground reaction force plates (P6000, BTS, Italy) at a sampling rate of 800 Hz.

Muscle activity was measured using an electromyographic system (FREEEMG, BTS Bioengineering, Milan, Italy). Specifically, surface EMG (sEMG) signals of the rectus femoris, biceps femoris, tibialis anterior, and lateral gastrocnemius muscles of both legs were measured at a sampling rate of 1000 Hz. For electrode placement, the Surface EMG for the Non-Invasive Assessment of Muscles (SENIAM) guidelines were followed [27]. For each side, ten gait cycles were collected and processed for further analysis.

### 2.5. Data Analysis

Three-dimensional marker trajectories of the 22 markers were tracked and reconstructed using a frame-by-frame tracking system (Smart Tracker, BTS Bioengineering, Milan, Italy).

Spatiotemporal parameters (stance, swing, single support and double support phases, step length, stride length, step width, mean speed, and cadence) and kinematic data (range of motion of the hip, knee, and ankle joints) were computed for both sides (the operated and the contralateral side), using dedicated software (SMART-Clinic, BTS Bioengineering, Milan, Italy). Regarding the kinetic data, i.e., joint moment and joint power, platform data were available only for a subset of patients who were not using walking aids during the assessment. Consequently, kinetic data were not included in the study.

The analysis of sEMG was performed using Matlab (Matlab R2023b, MathWorks, Natick, MA, USA). Raw sEMG signals were band-pass filtered (10–400 Hz, 2nd order Butterworth), full-wave rectified, and then low-pass filtered (4 Hz, 4th order Butterworth) to obtain the sEMG linear envelopes [28]. Furthermore, these muscle activation patterns were amplitude-normalized for each gait cycle to the peak activation that occurred during all gait cycles [29] and time-normalized to 100% of the gait cycle. Then, to analyze changes in muscle activation patterns during gait, the following sEMG parameters were computed, as proposed by Infarinato et al. [30]: the root mean square (RMS), which represents overall muscular activity, and the co-contraction (CC) index, reflecting the coactivation of agonist–antagonist muscle pairs (rectus femoris/biceps femoris and tibialis anterior/gastrocnemius). For the RMSs, we did not normalize the signal amplitude, as some patients exhibited no measurable activation during gait.

### 2.6. Statistical Analysis

Descriptive and inferential analyses were performed using SPSS (version 28, IBM, New York, NY, USA) and Matlab. Owing to the small sample size, a non-parametric test was used. Specifically, to assess changes in spatiotemporal parameters, ROMs, and sEMG parameters after the rehabilitation program, the Wilcoxon signed-rank test was used. For all the analyses, a *p*-value lower than 0.05 was considered significant.

## 3. Results

### 3.1. Demographic and Clinical Characteristics

Demographic and clinical characteristics of all the enrolled patients are reported in Table 1. The study population consisted of 10 men and 11 women, with a mean age of 61.1 years (range: 32–81). The tumor was located in the thigh in 95% of cases, distributed as follows: n = 12 in the antero-medial or antero-lateral region, n = 1 in the medial region, and n = 7 in the posterior region. Additionally, one case involved retroperitoneal tumors affecting the iliopsoas muscle and femoral nerve.

Patients underwent different demolitive and reconstructive surgical procedures: demolitive surgery without subsequent reconstruction (12 patients), demolitive surgery with reconstruction using local perforator flaps (2 patients), demolitive surgery with free flap reconstruction (2 patients), and demolitive surgery with functional free muscle transfer (5 patients). Furthermore, 7 patients received adjuvant therapies such as radiation therapy, chemotherapy, or hyperthermia.

The mean interval between surgery and the initial instrumented assessment was 57 ± 44 days (range: 5–219 days). A second assessment was conducted, on average, 42 ± 16 days (range: 21–84 days) after the first. The timing of the first assessment varied due to several factors, including (a) the occurrence of surgical complications that could significantly delay admission to the rehabilitation ward; (b) patients’ ability to walk in relation to the type of demolitive and reconstructive surgery, which has different recovery times for ambulation [16]; and (c) the condition of their dressings and wounds, which needed to be suitable for the safe application of electromyographic sensors. The variability in the interval between the two assessments was due to the above-reported reasons and patients’ discharge.

After the rehabilitation intervention, the modified Barthel Index showed a statistically significant improvement, with mean values increasing from 60.5 ± 17.2 to 90.7 ± 9.8 (*p* < 0.001).

### 3.2. Instrumented Assessment Results

#### 3.2.1. Spatiotemporal Parameters

**Figure 1:**
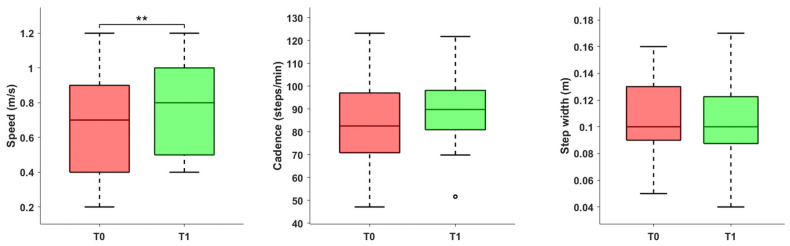
Spatiotemporal parameters (speed, cadence, and step width) before and after the rehabilitation treatment. Asterisks denote statistical significance, according to the Wilcoxon signed-rank test (** *p* < 0.01). The hollow circle represents an outlier.

As reported in Figure 1, following the rehabilitation program, an increase in both gait speed (*p* = 0.008) and cadence (*p* = 0.054) was observed. It is important to note that, in the case of walking speed, 16 patients exceeded the MCID for comfortable walking, defined as 0.1 m/s [31,32]. Among those who did not achieve this change, 2 patients already demonstrated a physiological walking speed exceeding 1 m/s at the initial evaluation.

**Figure 2:**
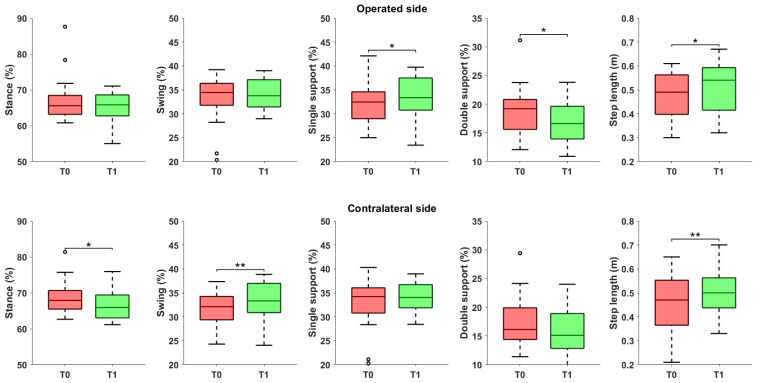
Spatiotemporal parameters referred to the operated and the contralateral sides, before and after the rehabilitation treatment. Asterisks denote statistical significance, according to the Wilcoxon signed-rank test (* *p* < 0.05, ** *p* < 0.01). Hollow circles represent outliers.

Significant changes were observed in the gait phases (Figure 2): specifically, on the operated side, we observed a reduction in the single support phase (*p* = 0.013) and the double support phase (*p* = 0.033). These changes were accompanied by significant variation in the contralateral limb, including a reduction in the stance phase (*p* = 0.011) and an increase in the swing phase (*p* = 0.008). Step length significantly increased on both the operated side (*p* = 0.016) and the contralateral side (*p* = 0.002). Additionally, in terms of asymmetry, a reduction in spatial and temporal asymmetry was detected, even though not statistically significant (*p* = 0.156 and *p* = 0.227, respectively).

**Figure 3:**
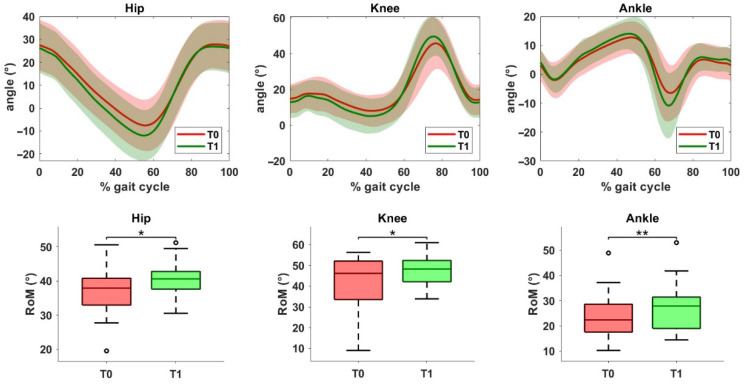
Kinematic data of the operated limb before (T0) and after (T1) the rehabilitation treatment. The top row shows joint angles of the hip, knee, and ankle in the sagittal plane. The solid line represents the mean value, while the shaded area indicates the standard deviation. The bottom row displays the range of motion (ROM) values for the same joints at T0 and T1. Asterisks denote statistical significance, according to the Wilcoxon signed-rank test (* *p* < 0.05, ** *p* < 0.01).

Finally, a statistically significant improvement was observed in sagittal plane range of motion for all three joints on the operated side (Figure 3). Specifically, increases were noted at the hip (*p* = 0.017), knee (*p* = 0.013), and ankle (*p* = 0.005).

#### 3.2.2. EMG Indexes

The statistical analysis of sEMG data comparing pre- and post-rehabilitation treatment on the operated side is detailed in Table 2 and Table 3. The findings reveal no statistically significant differences in the RMS values or the co-contraction indices. This lack of significant variation was consistent across all evaluated phases of movement, including the entire stride, the stance phase, and the swing phase. Similar data was obtained on the contralateral side.

## 4. Discussion

This study found that patients with lower-limb impairments after soft tissue sarcoma surgery showed significant improvements in walking ability after rehabilitation, as measured by instrumented gait analysis. This study, through a quantitative approach, reinforces the critical role of rehabilitation in improving gait characteristics in patients following surgery for soft tissue sarcoma. The data not only highlight the overall need for intervention but also provide valuable insights into the significant variability in patients’ conditions post-surgery. Moreover, these findings underscore the potential of quantitative metrics to guide increasingly personalized rehabilitation strategies, tailoring treatment to address the unique needs and challenges of each individual.

It is challenging to compare the results of this study with the existing literature on the topic, as, to the best of our knowledge, there are no similar studies that have evaluated the effectiveness of rehabilitation intervention in patients with lower-limb impairment following surgical treatment for soft tissue sarcoma using a quantitative assessment of gait pattern through gait analysis. The application of such methodologies in soft tissue sarcoma patients, particularly those with lower-limb impairment, remains scarce. However, the results of this study do confirm the positive effects of rehabilitation, as previously reported in the literature, although in very few studies, which typically rely on clinical scales. While these scales provide valuable information on overall improvement, they do not offer a detailed characterization of the changes in gait. In the study of Furtado et al. [23], gait characteristics of patients with soft tissue or bone sarcoma were compared to those of healthy subjects using an accelerometer-based sensor positioned on the lower back. The results revealed impairments in spatiotemporal parameters, including increased step time, stance time, and swing time, as well as reduced step length and step velocity, underscoring the necessity for personalized rehabilitation interventions.

As observed, rehabilitation plays a pivotal role in improving gait parameters in patients who undergo surgery for the resection of soft tissue sarcomas of the lower-limb or retroperitoneal soft tissue sarcomas with femoral nerve impairment [33]. Although patients with sarcoma often require intensive rehabilitation, there is a notable lack of disease-specific approaches tailored to their needs. Instead, general principles from cancer and musculoskeletal rehabilitation are frequently adapted for this population [34]. The principles underlying physiotherapeutic interventions are rooted in histological, biological, and biomechanical rationales [35], which work in synergy to promote recovery and improve functional outcomes. These principles guide the rehabilitation process, ensuring that treatment is not only effective but also tailored to the unique needs of the patient. Post-surgical rehabilitation stimulates the healing of muscle, tendon, and bone tissues by enhancing local vascularization, improving the delivery of oxygen and nutrients, and accelerating the repair of damaged tissues [36]. This process is crucial in minimizing recovery times and ensuring the quality of scar tissue, which in turn supports functional restoration. Furthermore, rehabilitation exercises contribute to controlling inflammation [37] by promoting increased blood flow to the affected areas, facilitating the removal of waste products accumulated during the inflammatory response, and preventing delays in healing caused by the retention of harmful by-products. Following surgery, patients often experience joint stiffness, particularly in the hip, knee, and ankle. Physiotherapy addresses this issue by incorporating passive and active mobilization exercises designed to improve the range of motion, prevent contractures, and restore normal joint functionality [38]. Additionally, prolonged immobilization or the post-surgical period can lead to changes in muscle tone, which rehabilitation aims to correct by restoring the balance between agonist and antagonist muscles. Strengthening exercise, initially performed at low intensity and gradually increasing, is crucial for improving joint stability and movement efficiency without overloading the healing tissues or joints. Moreover, surgical interventions can disrupt the biomechanical alignment of the lower limb, affecting posture and gait [13]. Following that, a key objective of rehabilitation is to assist patients in regaining a normal gait pattern while minimizing compensatory movements. This objective is crucial as compensatory strategies, which often develop in response to pain or instability, can lead to further musculoskeletal issues and impede overall recovery. In particular, rehabilitation focuses on reestablishing gait symmetry, retraining proper step patterns, to avoid long-term unilateral loading, and preventing compensatory postural mechanisms that could lead to further musculoskeletal issues [39]. Lastly, rehabilitation is vital in preventing postoperative complications, musculoskeletal disorders, and the risk of falls, all of which are common concerns for patients recovering from sarcoma surgery [14]. By addressing these diverse aspects of recovery, rehabilitation ensures that patients not only regain their ability to walk but also enhance the quality and efficiency of their gait, ultimately leading to improved functional outcomes and quality of life.

In our sample, electromyographic signal analysis did not reveal statistically significant changes in the analyzed indices, namely the RMS of the signal and the co-contraction index. This finding appears to contrast with the observed improvements in spatiotemporal parameters. However, it is important to note that patients exhibited significant heterogeneity in terms of tumor location, severity, type of surgical intervention, and other clinical factors. Consequently, it is possible that the observed improvements in gait patterns were achieved through the adoption of different muscle activation strategies. It remains unclear whether these strategies are entirely patient-specific or linked to particular clinical characteristics. Nonetheless, the small sample size precludes the possibility of conducting subgroup analyses on motor strategies in relation to the side of the tumor and the kind of demolitive and reconstructive surgery. Future studies should investigate this topic in greater detail using larger cohorts. However, the rarity of this tumor, along with its diverse clinical presentations and the associated variability in surgical approaches, underscores the complexity of achieving this research goal. Furthermore, it is important to highlight that literature reports a wide range of indices derived from sEMG, each capturing different facets of muscle function and neuromuscular control. This raises the possibility that rehabilitation did not affect the RMS and co-contraction indices that were considered in the analysis. Further analysis of alternative indices, which may include measures such as muscle activation timing, additional time-series, frequency-domain features, nonlinear metrics, or complexity metrics, could potentially reveal subtle changes in muscle coordination, recruitment patterns, or fatigue resistance that are not apparent in the indices analyzed in this study. Exploring these additional metrics in the future could provide a more comprehensive understanding of the neuromuscular adaptations that occur during rehabilitation and help identify clinically relevant improvements that may otherwise go unnoticed. Future research should consider incorporating a broader spectrum of sEMG-derived indices to fully characterize the effects of rehabilitation on muscle function and movement quality.

The main limitations of this study are the small sample size, the lack of a comparison group, and the heterogeneity of patients in terms of tumor sites, characteristics, and surgical approaches. However, unlike other studies, it is important to note that our sample was homogeneous in terms of the diagnosis of soft tissue sarcoma (excluding bone sarcoma) and the surgical goal of limb salvage. Despite this, due to the rarity of soft tissue sarcoma, the sample size was too small to allow for meaningful subgroup analyses based on surgery type or tumor location. This limitation hindered our ability to explore how gait outcomes varied after rehabilitation across different tumor sites, characteristics, or surgical approaches, making it difficult to draw firm conclusions for specific patient subgroups. A further limitation is the variability in the timing of baseline assessments after surgery, which ranged from early to late postoperative phases. Given the exploratory nature of the study and the limited sample size, we opted for non-parametric paired analyses (Wilcoxon test), without adjustment for surgical delay, to avoid model overfitting and preserve interpretability.

Future studies with larger, more homogeneous cohorts would be beneficial to further elucidate the impact of specific treatment modalities and tumor characteristics on rehabilitation outcomes in this patient population. Moreover, they should aim to analyze the relative contribution of each joint to gait pattern differences in this specific patient population by integrating advanced approaches such as machine learning. This methodology has recently demonstrated significant potential in gait pattern recognition and clinical gait analysis, offering novel insights into the complex mechanisms underlying gait adaptations [40]. Such approaches could help to refine patient stratification and optimize targeted rehabilitation strategies

## 5. Conclusions

Using quantitative gait analysis, we observed significant improvements in spatiotemporal parameters and joint mobility, highlighting the crucial role of personalized rehabilitation programs, tailored to the specific type of demolitive and reconstructive surgery, in improving gait performance and functional mobility in patients with soft tissue sarcomas.

Although electromyographic indices did not show statistically significant changes in the whole group, this may reflect diverse and patient-specific motor strategies, influenced by tumor location and surgical approach.

These findings underscore the importance of objective assessment tools and personalized interventions in guiding recovery.Further insights into neuromuscular adaptations could enhance the development of more targeted rehabilitation protocols, ultimately improving clinical outcomes and quality of life for sarcoma survivors. Despite the limitations related to sample size and heterogeneity, this study contributes valuable evidence to a still limited field and supports the growing awareness of rehabilitation as a key element in comprehensive sarcoma care.

## Figures and Tables

**Table 1:** Demographics, tumor characteristics, surgical procedures, and medical treatment for each patient. F: female; M: male; L: left; R: right; FFMT: free functional muscle transfer; DS: demolition surgery; FF: free flap; LPF: local perforator flap; RT: radiotherapy; CT: chemotherapy; Ht: hyperthermia.

ID	Sex	Age	Tumor	Side	Type of Surgery	Medical Therapy	Gait Aid (Forearm Crutch) During the Instrumented Exams	Time from Surgery to Rehabilitation (Days)	Time Between the Two Evaluations (Days)
1	F	77	Pleomorphic sarcoma	L	FFMT	RT + Ht + CT	no	35	53
2	M	47	Liposarcoma	R	FF	CT	no	29	30
3	M	60	Pleomorphic sarcoma	L	DS	NA	yes	219	57
4	M	78	Atypical lipomatous tumor	L	DS	NA	yes	76	36
5	F	80	Myxofibrosarcoma	L	FFMT	RT + Ht	yes	91	53
6	M	72	Pleomorphic sarcoma	L	FFMT	RT + Ht	yes	65	27
7	M	57	Leiomyosarcoma	R	FF	CT	no	42	61
8	F	74	Liposarcoma	R	DS	NA	yes	56	35
9	F	77	Liposarcoma	L	DS	NA	no	32	63
10	F	45	Myxofibrosarcoma	L	FFMT	RT + Ht + CT	yes	55	21
11	F	51	Spindle cell sarcoma	L	DS	NA	no	58	84
12	M	49	Dedifferentiated sarcoma	L	DS	NA	no	5	26
13	F	47	Liposarcoma	L	DS	CT	no	73	30
14	F	54	Atypical lipomatous tumor	R	DS	NA	no	21	27
15	M	78	Atypical lipomatous tumor	R	DS	NA	no	20	36
16	F	60	Malignant peripheral nerve sheath tumors	R	LPF	NA	no	90	31
17	F	81	Small round cell	R	FFMT	NA	no	63	45
18	M	73	Atypical lipomatous tumor	L	DS	NA	yes	17	33
19	M	32	Myxofibrosarcoma	R	DS	NA	no	73	54
20	F	74	Hemosiderotic fibrolipomatous tumor	L	DS	NA	no	41	36
21	M	57	Leiomyosarcoma	R	LPF	NA	yes	43	41

**Table 2:** Co-contraction index (mean and standard deviation, SD) measured before (T0) and after the rehabilitation intervention (T1).

Variables	T0 (Mean ± SD)	T1 (Mean ± SD)	*p* (Wilcoxon Signed-Rank U Test)
CCI Shank			
*stride*	0.50 ± 0.09	0.51 ± 0.08	0.434
*stance*	0.52 ± 0.10	0.53 ± 0.11	0.875
*swing*	0.61 ± 0.11	0.63 ± 0.07	0.476
CCI Thigh			
*stride*	0.59 ± 0.07	0.59 ± 0.05	0.821
*stance*	0.61 ± 0.07	0.63 ± 0.06	0.566
*swing*	0.60 ± 0.07	0.57 ± 0.08	0.169

**Table 3:** Root mean square (RMS) (mean and standard deviation, SD), measured before (T0) and after the rehabilitation intervention (T1).

Variables	T0 (Mean ± SD)	T1 (Mean ± SD)	*p* (Wilcoxon Signed-Rank Test)
RMS Biceps femoris (µV)			
*stride*	41.5 ± 56.4	51.5 ± 66.3	0.258
*stance*	42.8 ± 57.2	51.5 ± 65.6	0.321
*swing*	37.8 ± 55.4	49.9 ± 68.5	0.159
RMS Rectus femoris (µV)			
*stride*	48.1 ± 51.9	44.3 ± 55.2	0.614
*stance*	54.3 ± 54.8	48.6 ± 53.7	0.339
*swing*	31 ± 46.3	32.1 ± 58.1	0.875
RMS Tibialis anterior (µV)			
*stride*	32.4 ± 37.6	30.8 ± 40.4	0.875
*stance*	37.4 ± 45.6	34.2 ± 46.4	0.663
*swing*	16.4 ± 15.4	20 ± 22.9	0.958
RMS Gastrocnemius lateralis (µV)			
*stride*	57.8 ± 33.5	55.6 ± 31.4	0.414
*stance*	56.4 ± 36.9	53.1 ± 33.1	0.273
*swing*	56 ± 28.4	55.9 ± 30.8	0.638

## Data Availability

The datasets used and/or analyzed are available from the corresponding author upon reasonable request.
